# Lack of Avidity Maturation of Merozoite Antigen-Specific Antibodies with Increasing Exposure to *Plasmodium falciparum* amongst Children and Adults Exposed to Endemic Malaria in Kenya

**DOI:** 10.1371/journal.pone.0052939

**Published:** 2012-12-26

**Authors:** Frances Ibison, Ally Olotu, Daniel M. Muema, Jedida Mwacharo, Eric Ohuma, Domtila Kimani, Kevin Marsh, Philip Bejon, Francis M. Ndungu

**Affiliations:** 1 Kenya Medical Research Institute, Centre for Geographical Medicine Research (Coast), Kilifi, Kenya; 2 University of Birmingham Medical School, Edgbaston, Birmingham, United Kingdom; 3 University of Oxford, Nuffield Department of Medicine, John Radcliffe Hospital, Oxford, United Kingdom; Johns Hopkins Bloomberg School of Public Health, United States of America

## Abstract

**Background:**

Although antibodies are critical for immunity to malaria, their functional attributes that determine protection remain unclear. We tested for associations between antibody avidities to *Plasmodium falciparum (Pf)* antigens and age, asymptomatic parasitaemia, malaria exposure index (a distance weighted local malaria prevalence) and immunity to febrile malaria during 10-months of prospective follow up.

**Methods:**

Cross-sectional antibody levels and avidities to Apical Membrane Antigen 1 (AMA1), Merozoite Surface Protein 1_42_ (MSP1) and Merozoite Surface Protein 3 (MSP3) were measured by Enzyme Linked Immunosorbent Assay in 275 children, who had experienced at least one episode of clinical malaria by the time of this study, as determined by active weekly surveillance.

**Results:**

Antibody levels to AMA1, MSP1 and MSP3 increased with age. Anti-AMA1 and MSP1 antibody avidities were (respectively) positively and negatively associated with age, while anti-MSP3 antibody avidities did not change. Antibody levels to all three antigens were elevated in the presence of asymptomatic parasitaemia, but their associated avidities were not. Unlike antibody levels, antibody avidities to the three-merozoite antigens did not increase with exposure to *Pf* malaria. There were no consistent prospective associations between antibody avidities and malaria episodes.

**Conclusion:**

We found no evidence that antibody avidities to *Pf*-merozoite antigens are associated with either exposure or immunity to malaria.

## Introduction

The anti-parasitic and therapeutic effects of passively transferred malaria immune IgG, from immune adults to children or adults with malaria, provided strong evidence that antibodies are critical mediators of naturally acquired immunity to malaria [1]–[3]. Subsequent sero-epidemiological studies to identify antibody specificities and mechanisms of immunity have produced conflicting results and have failed to reveal easily measurable correlates of protection [4], [5]. Conflicting results in sero-epidemiological surveys may result from confounding by exposure [5]–[7]. Furthermore, qualitative properties of antibodies such as isotype, avidity and functional activity may be as important as overall concentration. Previous studies have implicated cytophilic IgG1 and IgG3 antibodies in parasite killing mechanisms like opsonisation, phagocytosis and antibody dependent cellular inhibition (ADCI) that require cooperation with monocytes and macrophages via FcγRI and FcγRII [8], [9]. More recently, the breadth of antibody specificity has also been associated with immunity [10]. Antibody avidity is an important correlate for immune memory and protective immunity in other infections [11], [12] and vaccine trials [13], [14], but has only rarely been studied in malaria.

Achtman et al demonstrated increasing avidities of MSP1_19_-specific antibodies with multiple *P. chabaudi* infections in mice, suggesting that avidity maturation occurs in *Plasmodium* infections [15]. In agreement, Ferreira et al reported increased *Pf*-lysate-specific antibody avidities following resolution of clinical malaria in Brazilian adult patients, suggesting that human *Pf* infections are also associated with avidity maturation [16]. More recently, Leoratti et al demonstrated higher avidities among children with uncomplicated and asymptomatic malaria relative to children with complicated malaria [17]. Tutterow et al found that antibodies binding to VAR2CSA with high avidity were associated with reduced risk of placental malaria [18]. Reddy et al found that antibody avidities for AMA-1 and MSP2-3D7 increased with age, and that individuals with the highest antibody avidities for MSP2-3D7 at the baseline of a prospective study had a prolonged time to clinical malaria [19]. Together, these reports suggest that avidity maturation, at least to the antigens studied, is important in the development of naturally acquired immunity to malaria. In contrast, Akpogheneta et al observed no consistent associations of antibody avidities for several merozoite antigens with seasonal transmission patterns, age, asymptomatic parasitaemia, or occurrence of clinical malaria in Gambian children living in an area of low transmission [20].

In the present study, we tested whether cross-sectional antibody avidities (as well as antibody levels) to three *Pf*-merozoite antigens are associated with age, asymptomatic parasitaemia, distance weighted local malaria prevalence (EI) (hereafter referred to as malaria exposure index [21]) and prospective immunity to malaria in a large and well-characterized longitudinal cohort of Kenyan children living in an endemic area.

## Materials and Methods

### Ethics

This study was approved by the Kenyan Medical Research Institute National Ethics Committee. Written informed consent was provided by the parents/guardians of all the children involved.

### Study site

The study was done at the KEMRI Centre for Geographic Medicine Research Coast situated at Kilifi District Hospital, Kenya. The hospital serves ∼500,000 people living in Kilifi District. The children investigated in this study were part of longitudinal cohort of 300 resident in Junju location, lying on the southern side of an Indian Ocean creek that divides Kilifi into North and South. This study site is predominantly inhabited by the Mijikenda people [22], [23].

### Study population

Although there has been a gradual decline in *Pf* transmission in Kilifi district [24], [25], Junju remains stably endemic with two high transmission seasons (in May to August, and October to December) and a parasite prevalence of 30% [26], [27]. Children are recruited into Junju cohort at birth and actively followed weekly [26] for detection of malaria episodes (defined as an axillary temperature >37.5 degrees centigrade, with a *P. falciparum* parasitemia >2500 parasites per microliter) until the age of 13 years. We maintain extensive and detailed records of the numbers and dates of malaria experiences for each child, either from birth or from the time of recruitment.

### Plasma

5 ml venous blood samples and blood smears were collected in a pre-season cross-sectional survey in May 2009, a time preceded by four months of minimal *Pf* transmission in Junju. Plasma was harvested and stored at −80°C.

### Antigens

*Pf-*specific IgG antibody responses were quantified against recombinant *Pf* AMA1-FVO/3D7 (1∶1 mixture by weight of the two proteins (alleles)), MSP1_42_ and MSP3, to which circulating IgG antibodies have been associated with clinical protection in our study population [10], [28]–[30]. Recombinant *Pf* antigens were provided by Dr. Louis Miller (NIH, USA).

### Determination of parasitaemia

Thick and thin blood smears were stained with Giemsa and *Pf*-infected red cells counted against 500 leukocytes and 1000 red blood cells, respectively, by expert microscopists.

### Avidity

Antigens were coated onto flat-bottomed 96-well microtitre plates (MICROLON®) overnight at a concentration of 100 ng/ml in bicarbonate buffer. Coated plates were washed three times with Phosphate Buffered Saline (PBS) with 0.05% Tween (PBST), before blocking for 1 hour with 10% Foetal Calf Serum (FCS) in PBS.

The plasma samples were diluted to 1∶500 in 0.3% PBST with 1 mM Ethylenediaminetetraacetic acid (EDTA) and 100 µl was added to each of the four wells allocated to each sample. A doubling dilution series from 1∶250 to 1∶32,000 of a pool of hyper-immune plasma from local adults, in duplicate wells on each plate, was used to produce standard curves and as a positive control. Plasma from seven non-immune European donors, also in duplicate wells on each plate, was used to generate a cut-off and as a negative control. Plates were incubated with the test/control plasma at room temperature for 2 hours then washed five times. 100 µl of 4 M guanidine hydrochloride, prepared in 0.05% PBST, was added to two out of four wells for each plasma sample, with 0.05% PBST alone added to the remaining two wells. After 10-minute incubation at room temperature the plates were washed four times. Alkaline phosphatase-conjugated Goat anti-Human IgG (Fc-specific, Sigma®), plus substrate, were then used to assess the amount of IgG remaining bound in each well. Optical densities (OD) were read at 405/570 nm.

Test samples with a mean OD less than two standard deviations above that of non-immune European controls (for MSP3) or less than four standard deviations above (for AMA1 and MSP1) were classed as negative for antibody to that antigen, and avidity indices were not calculated. A sigmoidal standard curve from the doubling dilution series of pooled hyper-immune adult plasma was used to convert mean OD (without guanidine) into an antibody level in Arbitrary Units (AU). Avidity indices were calculated as the ratio of guanidine-treated mean concentration to non-guanidine-treated mean concentration, multiplied by 100.

### Exposure index

This was calculated as distance weighted proportion of malaria infections (both asymptomatic and symptomatic) within 1 km radius of each child [21]. Around 900 (including the 300 Junju-cohort) children living in the study area under active surveillance were used to estimate the individual exposure indices for the period prior to the cross sectional survey. For this analysis we calculated mean exposure index for each child covering the period from the start of follow-up to the cross sectional survey.

### Statistical Analyses

Log transformed antibody and avidity data were analyzed using Stata (Stata Corp, version 11) and GraphPad Prism for Macintosh (GraphPad Software, version 5.01). Multivariable linear regression was used to test the predictive value for age, asymptomatic parasitaemia, rates of previous clinical malaria attacks, and EI on levels and avidities. Associations between different continuous measures were determined by using linear regression. Wilcoxon rank-sum test was used where appropriate to compare median differences between groups for continuous variables. Associations between antibody responses (levels and avidities), age, and asymptomatic parasitaemia with the risk to the first (or only) episode of *Pf* malaria were determined by Cox regression analyses. Poisson regression models were fitted to determine whether the number of multiple malaria episodes were associated with antibody responses, age, and asymptomatic parasitaemia. For all tests, statistical significance was considered at the 5% level.

## Results

### Characteristics of study subjects

We tested samples from those children within the Junju cohort for whom we had documented evidence of at least one incident of malaria exposure since the start of surveillance in January 2005. From the cohort, 263 children had experienced at least one documented episode of clinical malaria by the cross-sectional sampling date in May 2009, rising to 275 children by the end of the follow up period 10 months later. The mean age at the sampling date was 6.2 years (standard deviation [SD] 2.46 years) (Table 1). The mean number of previous malaria episodes by sampling date was 3.27. The mean time elapsed between the last recorded episode and the sampling date was 11.4 months (SD 11.04 months). At the time of sampling, 45 children (16.4%) had asymptomatic *Pf* parasitaemia.

**Table 1:** Characteristics of the study subjects.

Sample size, number (No.)	275
Females: No. (%)	139 (50.6%)
Males: No. (%)	136 (49.4%)
Mean age (years) ± SD	6.18±2.46
*At least 1 previous episode: No. (%)	263 (95.6%)
*Mean number of previous episodes	3.27
*Number of previous episodes, range	0–12
*Mean time since last episode (months) ± SD	11.40±11.04
Asymptomatic parasitaemia at sampling - No. (%)	45 (16.4%)

* Previous episodes only refer those clinical malaria episodes that occurred before May 2009, the sampling date.

To be certain of substantial amounts of antibody, before attempting to determine avidity, we first screened all of the 275 plasma samples for the presence of antibody concentrations well above the mean levels of a panel of malaria-naïve control sera from the UK (see methods for positivity threshold). Of 275 children, 184 (67%), 218 (79%) and 130 (47%) children had antibody levels above the threshold of positivity for AMA1, MSP1 and MSP3, respectively. However, the chosen threshold for MSP3 antibody positivity was much lower than for AMA1 and MSP1 as only 29% of the children were positive at the mean + 4SD cut-off. These samples went on to be tested for antibody avidity indices. Antibody avidity indices were generally independent of the respective antibody levels (Figure S1).

Separate sets of samples were taken from 15 randomly selected children and 15 adults (“rural-adults”) from the same malaria-exposed village and from 8 adults (“urban-adults”) living in a semi-urban area of Kilifi with no *Pf* transmission. These samples were randomly selected from the May 2010 annual cross-sectional-bleed (of Junju children and 50 adults), taken following administration of written informed consent (as stated in methods section). Antibody levels and avidities to AMA1 and MSP1 were measured for comparison between the groups.

### Lack of avidity maturation with exposure to Pf for anti-AMA1, MSP1 and MSP3 antibodies

**Figure 1:**
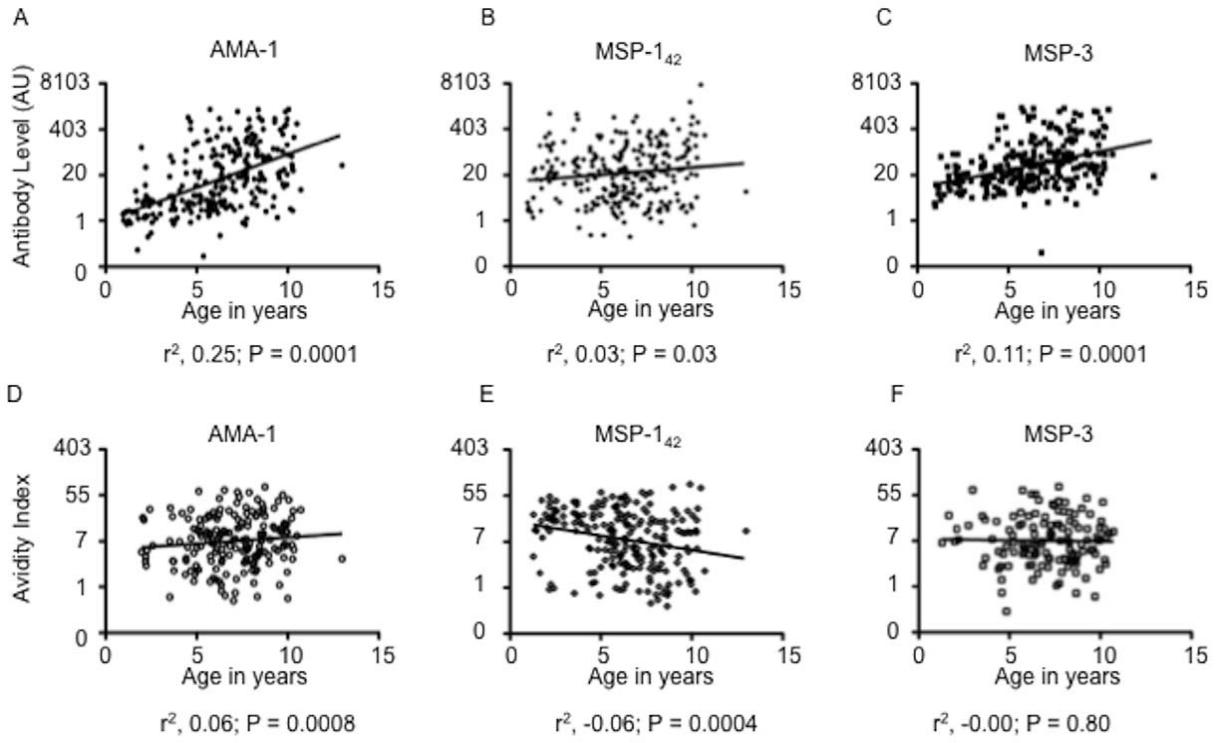
Relationships of antibody levels and avidities with age. Antibody levels and avidity indices for each antigen are plotted against age. Linear regression lines are shown, as are the associated R squared and P values.

Antibody levels to AMA1, MSP1 and MSP3 were positively correlated with age (Figure 1: A–C). In contrast, only anti-AMA1 antibody avidity showed a positive (but weak) correlation with age (Figure 1D). Moreover, anti-MSP1 antibody avidity was inversely (but weakly) associated (Figure 1E), while anti-MSP3 antibody avidity was not associated with age (Figure 1F).

To test whether the amount of previous malaria exposure may determine the cross-sectional levels and avidities of antimalarial antibodies, we checked for association with three markers of exposure: age, concurrent asymptomatic parasitaemia and the malaria exposure index (EI).

**Figure 2:**
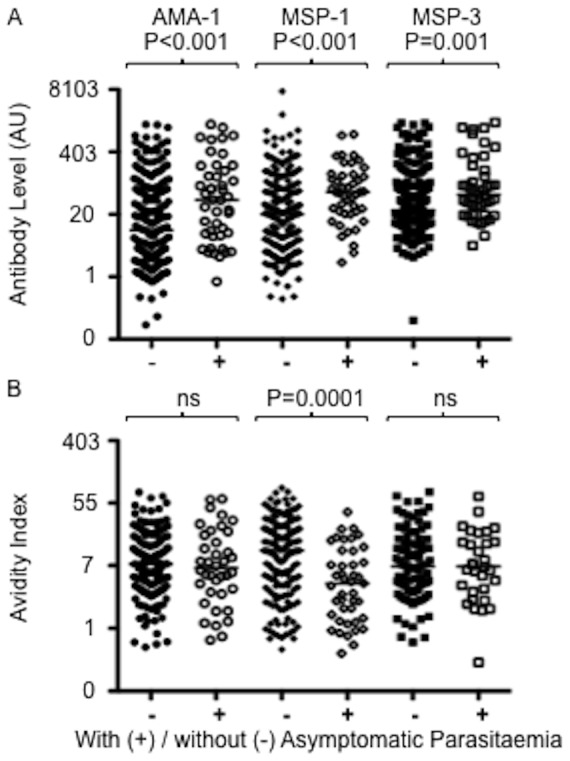
Antibody levels but not avidities were elevated in the presence of asymptomatic parasitaemia. Antibody levels (top panel) and avidities (lower panel) for children with (open symbols) and without (filled symbols) asymptomatic *P. falciparum* parasitaemia as determined by use of blood-films at sampling. Horizontal bars are medians. Statistical significance of differences was determined by Wilcoxon rank-sum test.

Antibody levels to all three antigens were significantly higher in children with asymptomatic parasitaemia compared to uninfected children (Figure 2A). In contrast, avidity indices were not positively associated with asymptomatic parasitaemia, and there was a significant negative association for MSP1 (Figure 2B).

We also used the distance weighted local malaria prevalence for each child as a marker of the total malaria exposure at the individual level as described in Olotu et al [21]. Antibody levels to each of the three-merozoite antigens increased positively with EI in multivariable linear regression models that controlled for age and asymptomatic parasitaemia at cross sectional survey (Table 2). In contrast, none of the three antigen-antibody avidities increased with EI, and there was a weak negative association for AMA1 and MSP1 (Table 2). Interestingly, the anti-AMA1 antibody avidity was not associated with age in this multivariable analysis in spite of the positive association in univariable analysis (Figure 1, above). Together these analyses suggest that whilst anti-malarial antibody levels are generally elevated with asymptomatic infections, these newly generated antibodies appear not to undergo significant avidity maturation. There is even a suggestion that parasite carriage may be associated with a reduction in antibody avidities, to a variable extent for different antigens. Additionally, whilst antibody levels to the three-merozoite antigens increase with EI, avidities do not.

**Table 2:** Multivariable analysis of association between EI, age and cross sectional parasitaemia with antibody levels and avidities.

		AMA1		MSP1		MSP3	
Antibody levels	Covariate	Coefficient (95% CI)	*P*	Coefficient (95% CI)	*P*	Coefficient (95% CI)	*P*
	EI	2.07 (0.90–3.24)	**0.001**	1.89 (0.65–3.07)	**0.003**	1.30 (0.43–2.17)	**0.003**
	Age (months)	0.04 (0.03–0.05)	**0.001**	0.01 (−0.001–0.01)	0.07	0.02 (0.02–0.03)	**0.001**
	Parasitaemia	1.05 (0.48–1.61)	**0.001**	0.94 (0.35–1.54)	**0.002**	0.73 (0.30–1.16)	0.001
Antibody avidities	EI	−0.36 (−1.32–0.60)	0.46	−0.67 (−3.14–1.48)	0.13	0.06 (−1.05–1.17)	0.9
	Age (months)	0.005(−0.002–0.01)	0.18	−0.02 (−1.57–−0.20)	**0.001**	−0.001 (−0.01–0.01)	0.8
	Parasitaemia	−0.42(−0.47–0.39)	0.85	−0.92 (−1.33–−0.50)	**0.001**	−0.03 (−0.51–0.46)	0.9

Data was analysed by multivariable linear regression.

### Comparison of avidity maturation for antibodies to Pf merozoite antigens and tetanus toxoid

**Figure 3:**
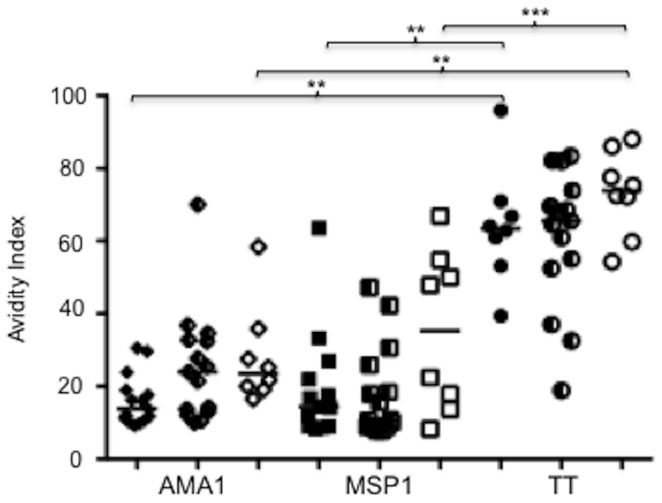
Comparisons of antibody avidities to AMA1, MSP1 and tetanus toxoid (TT). Children, rural-adults and urban-adults are represented by filled, half-filled and open symbols, respectively. Solid lines represents medians. Statistical significance between pairs of groups was determined with the Kruskal-Wallis test (* P<0.05, ** P<0.01, *** P<0.001).

To further explore the apparent lack of avidity maturation with malaria exposure, we compared anti-AMA1, MSP1 and tetanus toxoid (TT) antibody avidities from 15 randomly selected children, 15 rural-adults, and 8 urban-adults. Compared with the avidity indices for antibodies specific to the tetanus toxoid vaccine antigen, avidities for the two malaria antigens (AMA1 and MSP1) were significantly reduced, irrespective of age (Figure 3), suggesting that malaria infections are far less efficient at inducing avidity maturation to *Pf* antigens.

### Associations between AMA1, MSP1 and MSP3 -specific antibody avidities and subsequent malaria risk

We tested prospectively whether the antibody levels and avidities to AMA1, MSP1 and MSP3 measured after five months of minimal *Pf* transmission, but just prior to the subsequent two transmission seasons (10-months), were associated with the subsequent risk of malaria. For this analysis a malaria episode was defined as an axillary temperature of 37.5°C associated with *Pf* asexual parasitemia of over 2500 parasites per µl of blood. Two measures of malaria risk were analyzed: 1) time to the first, or only, malaria episode, and 2) incidence, including all episodes of malaria within the 10-month follow-up. The corresponding Cox and Poisson regression models controlled for age, EI and asymptomatic parasitaemia at the baseline. In multivariable analyses, both asymptomatic parasitaemia at the baseline and a higher EI were associated with shorter time to the first episode, as well higher incidence of clinical malaria (Table 3). However, the p values for the associations between asymptomatic parasitaemia and time to the first episode (p = 0.03) and multiple episodes (p = 0.02), and EI with time to the first episode (p = 0.01) are not significant after Bonferroni adjustment for multiple corrections (i.e. p = 0.18, p = 0.12 and p = 0.06, respectively). Likewise, high anti-MSP1 antibody levels were associated with increased incidence of malaria episodes in Poisson regression, but with no significant association with time to the first episode of clinical malaria. High anti-AMA1 antibody avidity was associated with delayed time to the first episode, but not with reduced incidence of all episodes of malaria in Poisson regression. The p values associated with these findings (i.e. p = 0.03 and p = 0.02, respectively) are not significant after a Bonferroni adjustment for multiple comparisons (i.e. p = 0.18 and 0.12, respectively).

**Table 3:** Prospective association between antibody levels and avidities at baseline with the risk of malaria during 10-months follow up.

	*Time to the first, or only episode*			*Multiple malaria episodes*		
Covariate	Hazard Ratio	95% Confidence Interval	P value	IRR	95% Confidence Interval	P value
AMA1 antibody level	0.97	0.81–1.16	0.72	0.97	0.89–1.06	0.51
**AMA1 antibody AI**	**0.72**	**0.56–0.94**	**0.02**	0.96	0.82–1.12	0.58
MSP1 antibody level	0.94	0.79–1.11	0.46	0.93	0.86–1.02	0.12
MSP1 antibody AI	1.02	0.85–1.24	0.81	**1.12**	**1.01–1.25**	**0.03**
MSP3 antibody level	0.96	0.76–1.21	0.73	0.93	0.80–1.07	0.32
MSP3 antibody AI	1.05	0.86–1.27	0.65	1.04	0.936–1.16	0.44
Age (months)	0.99	0.98–1.00	0.10	1.00	0.99–1.00	0.26
**Asymptomatic Parasitaemia**	**1.96**	**1.09–3.57**	**0.03**	**1.38**	**1.06–1.80**	**0.02**
**Exposure Index**	**3.16**	**1.41–7.07**	**0.01**	**1.98**	**1.33–2.95**	**0.001**

Summary of a multivariable Cox and Poisson Regression models for both Antibody levels and avidities, controlling for age, baseline parasitaemia and exposure index. AI, avidity index; IRR, Incidence Risk Ratio.

## Discussion

Sero-epidemiological studies have reported inconsistent associations between antibody levels to defined *Pf* antigens and naturally acquired immunity to malaria, leading to the hypothesis that qualitative and functional properties of antibodies may represent better correlates of immunity. Of the two main properties associated with the quality of antibody responses; avidity and isotype distribution, avidity is the least studied in malaria. We report a cross-sectional study investigating the effects of age, rate of exposure and immunity to malaria in children and adults living in an endemic area. We found that whilst antibody levels to all three merozoite antigens increased with age, the only antibody avidity to positively associate with age (albeit weakly) was for AMA1. However, this positive association was lost in multivariable analysis controlling for exposure and parasitaemia, suggesting that these two factors partially contributed to the association in univariable analysis. In contrast, anti-MSP1 antibody avidity was negatively, but weakly associated with age. Whilst antibody levels were elevated in the presence of asymptomatic parasitaemia, avidities were either reduced, in the case of MSP1, or unaffected, in the cases of AMA1 and MSP3. As expected from previous studies [21], antibody levels to all the three merozoite antigens tested were strongly associated with increasing exposure to *Pf*. In contrast, antibody avidity did not increase with EI. Furthermore, the associations between avidity and EI were negative for AMA1 and MSP1.

Classically, repeated exposures, with associated rounds of somatic hypermutation, are believed to pave the way for selection of memory B (and plasma) cells secreting antibodies of high avidity [31]. Hence the apparent lack of avidity maturation with increasing age (for MSP1 and MSP3) and exposure (for all three antigens) that we report here appears to contradict this classic dogma. In addition, we found higher avidities for the tetanus toxoid vaccine antigen than for the malaria antigens in both children and adults, suggesting that the extent of anti-merozoite antigen antibody avidity maturation is far less. This contrast is significant, given that the majority of the children tested here had only been immuninized to tetanus toxoid in three doses (maximum) as young children, but had been multiply infected with *Pf* parasites (up to 12 recorded malaria episodes for some). Furthermore, the negative association between MSP1 antibody avidity and asymptomatic parasitaemia suggests that asymptomatic infections may interfere with avidity maturation.

Few studies have associated antibody avidities with varying numbers of previous malaria episodes in humans. Reddy et al [19] and Leoratti et al [17] reported increasing avidities to malaria-specific antibodies with age and numbers of previous clinical malaria attacks, respectively. In addition, Ferreira et al (1996) demonstrated increasing IgG antibody avidities in adults recovering from acute malaria [16]. However, it is not clear whether such antibodies that have undergone avidity maturation during convalescence are maintained over time. Our results are in agreement with Akpogheneta et al, who reported increasing mean antibody levels, but not mean antibody avidities to the *Pf* merozoite antigens AMA1, MSP1_19_ and MSP2 from the pre-season baselines following the transmission season in the Gambia [20]. Collectively, these data suggest that while increased exposure to malaria is associated with higher antibody levels, the B cells that secrete these antibodies do not undergo avidity maturation. In support of this hypothesis, previous histological and immunohistochemical studies on the spleens of mice with acute malaria [32]–[35], and humans that died of severe malaria [36], have reported alterations in splenic structure and leukocytes that may affect normal germinal centre formation, where somatic hypermutation and selection of affinity matured B cells takes place [33]. Alternatively, highly avid antibodies could be produced in convalescence following parasite clearance in acute infections, but decay relatively fast thereafter. Further studies investigating the natural evolution of antibody avidity in children and adults recovering from *Pf* malaria will be required to clarify these issues.

In our study, there were no consistent associations of antibody levels and avidities with the risk of malaria. However, baseline asymptomatic parasitaemia and high EI were associated with increased risk of clinical malaria. Few other studies have attempted to associate avidities for *Pf* antigen specific antibodies with immunity to malaria. Akpogheneta et al did not observe any associations between avidities for pre-existing antibodies and subsequent occurrence of clinical malaria [20]. However, Leoratti et al demonstrated higher antibody avidities to total *Pf* antigen among children with uncomplicated and asymptomatic malaria relative to children with complicated malaria, suggesting that the ability to make antibodies of high avidity in response to infection may be associated with immunity to severe malaria. Tutterow et al found that antibodies binding to VAR2CSA with high avidity were associated with reduced risk for placental malaria [18]. Reddy et al showed that individuals with the highest antibody avidities for MSP2-3D7 at the baseline of a prospective study had a prolonged time to clinical malaria [19].

Because we controlled for heterogeneity of exposure to *Pf* parasites in our analysis, it is unlikely that it is the only possible explanation for the lack of association between antibody levels and immunity to clinical malaria. However, as we reported previously [37], antibody levels to malaria antigens are lost fairly quickly in the absence of chronic or persisting infections. Since the sampling for our study was done at the end of a 4/5-month dry season, it is likely that antibody levels in a substantial proportion of the children tested had waned to below the thresholds of protection.

We conclude that, in contrast to antibody levels, persistent exposure to *Pf* malaria does not result in avidity maturation for the anti-merozoite antigens AMA1, MSP1 and MSP3. Instead, our data suggests that persistent exposure to malaria, or increased prevalence of asymptomatic infections, may interfere with avidity maturation. Alternatively, it may be that antibody avidity maturation takes place quickly and plateaus after the first few infections, making it difficult for longitudinal studies to observe maturation with increasing exposure. Significantly, neither the anti-malarial nor anti-TT antibody avidities were different between children and adults. More studies are required to determine to what extent exposure to *Pf* malaria interferes with avidity maturation, and how this phenomenon is associated with naturally acquired immunity to malaria. A greater understanding of what it takes to induce protective antibodies remains a critical technological challenge in the struggle to develop highly efficacious antimalarial vaccines.

## Supporting Information

Figure S1 Avidity index is independent of antibody-levels. The avidity indices for A) AMA1 B) MSP1 and C) MSP3 were plotted against their respective antibody levels. The level of correlation was tested by linear regression analysis.
(TIFF)
