# Supplementary figures and images for: Lack of Avidity Maturation of Merozoite Antigen-Specific Antibodies with Increasing Exposure to Plasmodium falciparum amongst Children and Adults Exposed to Endemic Malaria in Kenya
Source: PLoS One. 2012 Dec 26;7(12):e52939. doi: 10.1371/journal.pone.0052939 (PMC3530478; doi:10.1371/journal.pone.0052939)

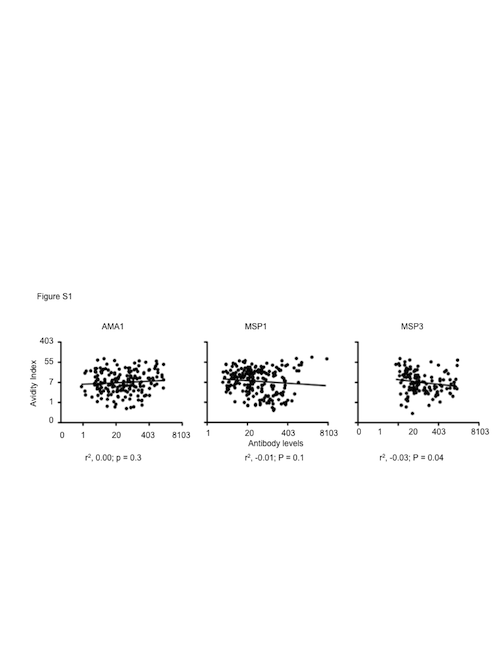

Supplement: Figure S1 — Avidity index is independent of antibody-levels. The avidity indices for A) AMA1 B) MSP1 and C) MSP3 were plotted against their respective antibody levels. The level of correlation was tested by linear regression analysis. (TIFF) [file pone.0052939.s001.tiff]
